# Commentary on: Practice Management Knowledge Amongst Plastic Surgery Residents in Canada: A National Survey

**DOI:** 10.1093/asjof/ojaa042

**Published:** 2020-09-23

**Authors:** Justin M Sacks

In the article titled Practice Management Knowledge Amongst Plastic Surgery Residents of Canada: A National Survey,^1^ the authors perform a critical survey assessing the knowledge of business and practice management’s core principles among plastic and reconstructive surgery trainees. The study assesses competency in the 9-core principles defined by Zarrabi et al^2^: healthcare marketing, business operations, human resource management, negotiation, insurance medical law, coding and billing, medical record management, finance, and accounting. The results of the survey had 2 important findings. First, respondents reported a strong desire for future training in business and practice management. Second, very few participants reported receiving any business training throughout their residency. Thus, the survey revealed both a desire for education in the competencies related to business practice and management and also a gap in educational knowledge in the Canadian plastic surgery training system.

Owing to the scope and complexity of practice, training to become a plastic surgeon is a long process that can take from 6 years to a decade—when accounting for dedicated research time and fellowship training. In this author’s experience, in an American-based plastic surgery program, there is a paucity of objective formalized business practice management education at the resident level. Residents with a specific interest or desire to learn about business practice must seek it out informally, while concurrently attending to their clinical duties. A rigorous curriculum in business and practice management has not been standardized across the American plastic surgery landscape. This survey succinctly provides a glimpse into a knowledge gap expressed by plastic surgery residents in Canadian plastic surgery training programs.

Studying healthcare financial management represents an opportunity to train plastic surgery residents to properly optimize their entry into academic, private practice, and hospital-based jobs. Financial management is a decision science.^3^ Gaining an understanding of business and practice management principles during the residency will prepare graduates to handle the challenges of clinical medicine that lie outside the operating room. It may help graduates with salary negotiation or to build a practice. This survey demonstrates that residents in the Canadian residency system report a lower level of knowledge and confidence in business and practice management. Despite a 50% (65/126) response rate, it was clear that a deficiency exists in the educational curriculum. One possible explanation may be that with increased requirements for programs to assess objective clinical milestones and decreased resident work hours, there is simply no space in the curriculum to train residents in nonclinical applications such as business and practice management.

Microeconomics can be defined as the allocation of scarce resources. Healthcare in the 21st century could not be more appropriately described. Reduced compensation for reimbursable clinical procedures compounded by escalating healthcare costs incurred by increasing salaries and costly new technologies creates an environment where understanding efficient practice management will become advantageous. Plastic surgery training programs have an opportunity to prepare trainees to address these challenges. The survey in this paper identifies a potential knowledge gap in plastic surgery residency training in North America. In this author’s experience, training in an academic program in the Northeast, and being exposed to academic and private practitioners, there was a lack of formal training in business and practice management. This problem is not restricted to the Northeast: personal experience in the southwestern and midwestern regions has demonstrated a similar pattern.

In the 2010 *Harvard Business Review* article, Turning Doctors into Leaders by Thomas H. Lee,^4^ the author mentions that healthcare’s new leaders must organize doctors into teams and measure their performance, not by how much they do but by how their patients fare. The author further expanded that we need to deftly apply financial and behavioral incentives, improve processes, and dismantle dysfunctional processes. A decade ago, this was a call to arms for physician leaders. A decade later, the results of this survey indicate that we can further improve our training of plastic surgeons to become healthcare leaders. While it is true that informal training, initiative, and intelligence may propel our trainees to become healthcare leaders irrespective of a formal business education, the survey indicates that we have the opportunity to better equip our graduates to navigate clinical medicine after their training. It is our duty to train competent physicians, and we must not neglect this essential competency.

The increase of the market capitalization of private healthcare companies and governments’ expansion into healthcare over the past decade puts healthcare management and the understanding of the business of medicine at the forefront. Solely focusing on clinical education will ill-equip surgical residents to take handle the multifaceted challenges of managing a practice and make appropriate clinical decisions. A formalized business of medicine academic curriculum integrated into plastic surgery training will be extremely beneficial for graduating residents entering into clinical practice, regardless of the financial model.

This survey initiated out of Canadian training programs, in my opinion, mirrors the educational training here in the United States regarding practice management. Increasing healthcare costs and pressure to graduate residents with demonstrable, objective clinical competencies in a shorter amount of time will create a necessity for training programs to improve their academic curriculum. It is a tremendous opportunity for the Plastic Surgery specialty to reexamine our educational curriculum and prioritize the development of critical skills in business and practice management among our trainees. National societies have previously tried and failed to address this gap in the curriculum. The inability to implement this curriculum change informally suggests that residency programs in North America ought to adopt a standardized business and practice management curriculum. This may improve healthcare delivery, quality, and efficiency long term. Direct applications of a business and practice management curriculum include: the allocation of scarce resources; improve the management of both private and academic plastic surgery practices; and will ultimately improve patient satisfaction, career satisfaction, and an improved plastic surgery society. This paper’s authors deserve recognition for asking this very important question: are we adequately preparing graduating residents to take on business and practice management? The answer to this question was obvious: we can do better, and we will.
